# Professional identity of Wuhan and Hong Kong social workers: COVID-19 challenges and implications

**DOI:** 10.1177/1473325020973339

**Published:** 2021-03

**Authors:** Yan-zhi Du, TM Simon Chan

**Affiliations:** College of Law and Political Science, Zhejiang Normal University, Zhejiang, China; Department of Social Work, Hong Kong Baptist University, Kowloon, Hong Kong

**Keywords:** Social work practice, social work education, China, Hong Kong, professional identity, Coivd-19

## Abstract

This reflexive essay focus on how COVID-19 has impacted the professional identity of social workers in Wuhan and Hong Kong. Exploratory and reflexive in nature, eight Wuhan social workers who comprised three males and five females, and seven Hong Kong social workers who comprised one male and six females were recruited for semi-structured interviews. Their experience in Wuhan and Hong Kong during COVID-19 were highlighted. The challenges to professional identity were analyzed and the reflection is categorized into four levels, namely, individual, community, educational and conceptual level. In sum, Wuhan interviewees were more struggled with educating the public on the difference between community work, volunteering and social work, especially at the hospitals, to protect the integrity of the social work profession which shows their commitment to their professional identity. Moreover, they found it difficult to position themselves in proactive online services, where hundreds of workers from different parts of the Mainland China would be involved. On the other hand, Hong Kong interviewees were more inclined to prioritize professional principles at levels that are even higher than those in standardized guidelines. Their goal is to take the best interests of their clients into consideration, and their self-reflections tend to focus more on professional judgement and development of the social work field, to pave the way for future enhancements. Finally yet importantly, the deficiencies of their education as evidenced by the pandemic have been made alarmingly explicit.

The focus of this reflexive essay is how COVID-19 has impacted the professional identity of social workers in Wuhan and Hong Kong, and the implications on social work practices and education in China will also be discussed.

## Professional identity and development in social work context

Professional identity is defined as self-perception within the context of an occupational profession, how this perception is communicated to others, and the “sense of being a professional” (Paterson et al., 2002). Professional identity also refers to a clear understanding of the related beliefs, attitudes and standards of being a member of a profession. As such, professional identity influences professional values, judgement and reflective practices (Pettifer and Clouder 2008). Obviously, there is a need for social workers in both Wuhan and Hong Kong to address COVID-19 in their practice settings, therefore it is worth discussing their experiences especially in terms of professional values and attitudes.

Wuhan is the capital city of Hubei Province and its largest city with a population of over 11 million people. In December 2019, the first case of COVID-19 emerged in Wuhan. Wuhan was subsequently placed under lockdown for nearly two months to contain the virus. On April 8, 2020, the lockdown in Wuhan officially ended. As of late June 2020, Hubei confirmed a total of 68,135 COVID-19 cases, including 50,340 in Wuhan and the provincial death toll stood at 4,512, including 3,869 in Wuhan.

Aside from Wuhan, Hong Kong was among the first group of Chinese cities that declared confirmed cases of COVID-19. Even this cosmopolitan city cannot avoid the spread of coronaviruses and their outbreaks. In 2003, the severe acute respiratory syndrome (SARS) appeared in Hong Kong, 1755 people became infected and 299 deaths.

## Data collection

Exploratory and reflexive in nature, eight Wuhan social workers who comprised three males and five females, and seven Hong Kong social workers who comprised one male and six females were recruited for semi-structured interviews, based on the inclusion criteria that they had provided direct social services to their residents and/or clients during the peak of COVID-19 in Mainland China from January to April 2020. The work experience of the Wuhan respondents as a social worker ranges from 5 to 12 years whilst that of their Hong Kong counterparts ranges from 4 to 20 years. The semi-structured interviews were done online or on the telephone because the lockdown of the cities at the time was not conducive to face-to-face contact, and lasted between 60 and 90 minutes. The first author, who interviewed the participants in Wuhan, received social work training in Wuhan and has more than ten years of work experience in both the tertiary education sector and social work field. The second author, who was responsible for the data collection in Hong Kong, received social work training in Hong Kong and the United Kingdom, and has more than thirty years of work experience in both the tertiary education sector and social work field. The interviews explored the views and perceptions of the participants around three topics: 1) experiences with providing services during the peak COVID-19 months; 2) challenges during the period of interest to their professional identity, if any; and 3) the efforts that they made to cope with the challenges to their professional identity during the height of the COVID-19 pandemic. The transcription of the audio-record was validated after the team of researchers concurred the text with the content analysis results. Nvivo-10 was used to complete the thematic analysis.

## Experience during COVID-19

In the face of the sudden changes demanded of the local community, the social workers of both regions had difficulties in adjusting to the “panic” mode of the community in response to COVID-19, such as the empty shelves in the grocery shops, and shutting down of offices and schools. They had to adjust to virtual means of contact instead of more personal and professional face-to-face meetings with their clients.

There are three types of services Wuhan social workers offered to their clients during the height of the pandemic. First, the university social work faculty staff, counselors, medical personnel and social workers offered virtual online counseling support and guidance; plus collaboration with medical personnel, volunteer and community workers, such as those in mobile cabin hospitals (武漢方艙醫院). Second, community workers, volunteers and social workers coordinated the tangible resources, such as food and medicine, virus prevention activities and duty rotation. Lastly, the social workers themselves volunteered to provide complimentary transportation, delivery and emergency services for that in-need during the lockdown.

On the other hand, the Hong Kong social workers stated that COVID-19 has had negative impacts on the families of their region; in particular, explicit increases in incidents of family conflict and violence, and collective depression and panic among family and clan members. On the flip side, some reported that the lockdown of the city gave self-isolating social service recipients and their family members the unexpected opportunity to spend more time together, which is a luxury to the people in rapid paced Hong Kong.

## Rethinking professional identity

### Revisiting and reconfiguring professionalism: Professional identity at the individual level

Social workers often need to revisit their professional identity after undergoing new experiences, which are related to changes in the environment, or organisation and practice settings. Since professional identity influences professional values and judgement (Pettifer and Clouder, 2008), both the social workers in Wuhan and Hong Kong shared the same challenges during COVID-19 but differing in idiosyncrasies.

The Wuhan social workers volunteered their time to provide online services and deliver resources. During these unprecedented times, they understood the need to adjust their role and focus on a wider variety of remote counselling services and health information provision. This dynamic shift in their role is manifested in the operations of sixteen mobile cabin hospitals (武漢方艙醫院), through which multi-disciplinary collaboration was carried out. The Wuhan social workers understood that the coordination of so many different types of personnel with limited resources would be challenging which prompted them to self-reflect on their professional identity, especially in terms of their degree of autonomy and role when they compared themselves to the community workers and volunteers. Similarly, while the Hong Kong Social Welfare Department announced special arrangements for subsidized welfare services and services, most of the study respondents in Hong Kong decided to offer additional and tailored services to fit the needs of their clients on a voluntary basis, such as collecting and delivering personal protective equipment and proactively developing online and remote services.

In addition, the pandemic posed challenges to the values, professional judgment and attitudes of the social workers at both the macro and micro levels. The majority of the Hong Kong interviewees were more inclined to prioritize ethical principles, like pursuing social justice, using a client-centered approach, showing empathy and maintaining confidentiality, at levels that are even higher than those in the standardized guidelines. Some questioned whether dogmatic practices would be able to meet the needs of their clients, while others wondered whether the degree and extent of the provided services are adequate enough, and still others compromised in working with other professionals, especially those who worked in the secondary settings. In sum, they have adopted an open and adaptive attitude in facing challenges and addressing their own shortcomings. On the other hand, the social workers in Wuhan focused on two factors. One is recognizing the limits of their professional skills when providing services and understanding their professional value. Therefore, they sought to find ways that would improve their knowledge and skills by continuously learning how to adapt to the needs of their clients. They placed their clients at the center, so as to enhance personal connectedness and professional mindfulness, by adhering to professional values, and reinforcing their professional identity. The second factor is multi-team cooperation with a large number of clients. That is, the social workers in Wuhan tried to effectively work together as a team but also manage challenges while maintaining their professionalism.

Based on the interviews, the discrepancy between the insights of the two groups and the problems that they faced could be explained by: (1) the level of stress that both the social workers and their clients faced with regard to the number of deaths, (2) the degree of public recognition of the social work profession, (3) the development and advancement of the social work profession and education in the two regions, and (4) the strategies used by the local authorities to control and reduce the impacts of COVID-19.

### An on-going process: Professional identity at the community level

The new normal during an ongoing pandemic has reshaped the professional identity of the social work community as a whole. Perhaps the problem is not with the pandemic *per se*, but the fact that as a phenomenon, COVID-19 calls for a reassessment of policies and measures so that the workers are better prepared.

In Wuhan and the neighbouring regions, the government has directed the anti-virus tasks in a well-organized but strict manner to coordinate the multi-disciplinary personnel. Wuhan social workers provide online services, as well as some direct services, including services as volunteers and community investigations with community workers. Social workers who do not offer online services are responsible for some of the practical services of their counterparts who are delivering virtual services. Although the practical services are limited due to difficulties in allocating resources and inaccessibility of some of the communities, there are still direct services offered. Nevertheless, some of the social workers had to self-quarantine during the height of this pandemic. In doing so, they felt an explicit sense of helplessness, not only due to the inability to deliver services but also the lack of service coordination with other helping personnel. How should they deliver services in a locked down city? How could they integrate both online services and arrange for delivery of tangible resources on a daily basis? How should they respond to the needs and concerns of the local residents amidst the chaos of a community lockdown?

In February and March 2020, the Hong Kong social workers struggled to decide on whether the non-governmental organizations should follow the practices of the government departments and close down all of their services. This would mean that most of the immediate services that intervened with family conflict, such as family violence, and also mental health related issues, like depression and suicidal cases, would not be offered and there would be no appropriate intervention offered. Despite the varying levels of involvement, the Hong Kong social workers generally believe that social workers should be tasked with addressing and handling the immediate needs of their clients during the global pandemic.

The self-reflections of the Hong Kong interviewees tend to focus more on professional judgement and development of the social work field, with concern on finding the means and resources to improve services in the future. In summarizing their experience as a whole, they recognize the importance of teamwork and supervision, which are also common areas of concern in the two regions. The scale of the teamwork among the social workers in Wuhan is however much larger, mainly because of its larger geographical size and population, and therefore the number of COVID-19 cases. Different institutions and agencies with different types of expertise, such as medical and social services in the local community, and the regional and provincial jurisdictions of both Hong Kong and Wuhan sought to collaborate in order to address the challenges. The Wuhan interviewees, who also appreciated the mentoring of their supervisors, emphasized the challenges around their professional identity during their self-reflection exercise. With regard to their reflections of the social work profession, several of the Wuhan interviewees would like the social work community to have a more distinct identity than the other helping professionals, so that the social worker identity would more resonate with the public.

### A valuable lesson: Professional identity at the educational level

The scale of the COVID-19 pandemic has offered new perspectives on the development of social work into a sustainable profession through education. One area that was the most striking for both the Wuhan and Hong Kong social workers during their self-reflection exercise is that they both recognize the deficiencies in social work education, including inadequate practice-based training opportunities and the lack of association between theoretical knowledge and real life cases.

Both the Hong Kong and Wuhan social workers reflected on their training and education which resulted in two lines of thought. While some felt helpless and frustrated about the shortcomings of the social work curriculum which has not prepared them for such a pandemic, others were reminded of the need for enhanced professional development in the long run on online platforms. However, sometimes challenges also present opportunities. Some of the participants stated that now is the time to extend the parameters of social work to include the more prevalent use of online education platforms, conduct online case work and family services, and even offer online group sessions.

Their education deficiencies as evidenced by the pandemic have been made alarmingly explicit. As such, how should tertiary institutions in both regions tailor their curriculum to address the challenges faced by the communities in the ‘new normal’? The professional body also has the responsibility to provide post-qualification training in the meantime to meet the diversified demands of both regions. If building a professional identity can be done in three ways, namely, through practice based training (Bruno and Aversana, 2018; Dorasamy and Rampersad, 2018), reflective learning (Rué et al., 2013; Threlfall, 2014), and diversified learning programs (Trede et al., 2012), post qualification training can then be advanced by considering these three factors.

### A relevant question: Professional identity at the conceptual level

Identity is not static, but fluid as it is constantly changing and modified in different interactive relationships. Professional identity is shaped by culture and society. Therefore, social workers situated in different regional cultures and environments offer diversity and enrich current understanding of the purpose of the social work field. Yet there might be confusion when social workers define their professional identity. It would be interesting to find out the ways through which their identity is produced. How would self-identification influence the professional development of social workers? What can be done to manage the impacts?

The relationship between the social worker and the self comes from the discrepancy between their own expectations and their actual behavior, which is reflected in their own professional competence. This concept is often fluid, as different individuals will have different interpretations about their own expectations and practices, which will produce different results, such as degree of satisfaction, frustration, or self-reconciliation.

The relationship between social workers and others comes from interaction with clients, co-workers, and supervisors which all have an impact on their identity. Positive feedback can acknowledge and reinforce their ability to self-identify as a good social worker, whereas negative feedback will have the opposite effect. At the same time, the support that comes from meaningful interactions with co-workers and supervisors can instill a sense of belonging and inclusion. These supports can help social workers construct a positive self-identity.

Furthermore, the relationship between social workers and their community is impacted by their interaction with those who have multi-specialties, understanding of different policies, degree of social recognition, and so on and so forth. The formation of an identity is affected by social attributes, and therefore formed and constructed within the social context; for example, the division of labor and interaction with other professionals in multi-team work (community workers and psychological counselors) or the public recognition of social work professionals, etc. For instance, the identity of social workers in Wuhan has been significantly enhanced with the continuous increase in government support of the social work profession.

As such, there is an ontological issue at stake globally, and we must rethink the professional identity of social workers. What are the values that social workers must uphold when lives are at risk? How can the personal and professional identities be reconciled? The pandemic therefore challenges a rethinking of how social work practices and education should be approached, in terms of how social workers engage with their professional identity amidst this global crisis.

There is no answer as yet and these questions are offered to all social workers globally. In order to better understand the professional identity of social workers in relation to the COVID-19 outbreak, understanding their experiences against a variety of different cultures is imperative. If we are to better equip our frontline workers, educators and learners, we need to be better informed of the diversity of the professional identity of social workers in other cultures and communities.
